# A proof of concept to define the parameters affecting poly-l-lactide-co-poly-ε-caprolactone shape memory electrospun nanofibers for biomedical applications

**DOI:** 10.1007/s13346-022-01218-2

**Published:** 2022-08-17

**Authors:** Silvia Pisani, Ida Genta, Tiziana Modena, Rossella Dorati, Giovanna Bruni, Marco Benazzo, Bice Conti

**Affiliations:** 1 Department of Surgical Sciences, Otorhinolaryngology Unit, Fondazione IRCCS Policlinico San Matteo, 27100 Pavia, Italy; 2 Department of Drug Sciences, University of Pavia, 27100 Pavia, Italy; 3 Department of Chemistry, Physico-Chemical Section, University of Pavia, Via Taramelli 14, 27100 Pavia, Italy

**Keywords:** Shape memory polymer, Electrospinning, Nanofibers, Design of experiment, Poly-l-lactide-co-poly-ε-caprolactone

## Abstract

This study is a proof of concept performed to evaluate process parameters affecting shape memory effect of copolymer poly-l-lactide-co-poly-ε-caprolactone (PLA:PCL) 70:30 ratio based nanofibrous scaffolds. A design of experiment (DOE) statistical approach was used to define the interaction between independent material and process variables related to electrospun scaffold manufacturing, such as polymer solution concentration (w/v%), spinning time (min), and needle size (Gauge), and their influence on Rf% (ability of the scaffold to maintain the induced temporary shape) and Rr% (ability of the scaffold to recover its original shape) outputs. A mathematical model was obtained from DOE useful to predict scaffold Rf% and Rr% values. PLA-PCL 15% w/v, 22G needle, and 20-min spinning time were selected to confirm the data obtained from theoretical model. Subsequent morphological (SEM), chemical-physical (GPC and DSC), mechanical (uniaxial tensile tests), and biological (cell viability and adhesion) characterizations were performed.

**Graphical abstract:**

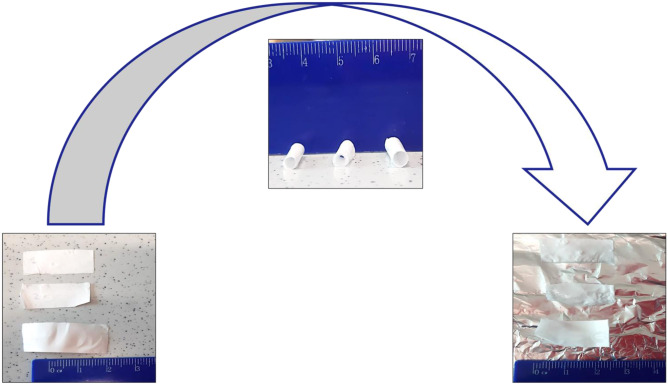

## Introduction

Shape memory polymers (SMPs) are emerging as new smart materials suitable to support biomedical applications. They are able to modify size, shapes, stiffness, or strain in response to different external (heat, electric and magnetic field, water, or light) and/or physiologic (pH, body temperature, and ion concentration) stimuli [1]. SMPs have the main ability to maintain an induced temporary deformation and recover their original shape at the end of the trigger exposure. This ability is allowed by a well-defined SMP chemical architecture characterized by the concomitant presence of molecular switching segments (sensible to a stimulus acting as reversible phases) and net-points (hardest support portion that determines fixed shape) [2].

SMPs can carry out different shape memory effects (SMEs), namely one-way (OWSME), two-way reversible (TWSME), and multiple SME. OWSMEs are characterized by a single opportunity to change shape; in fact, once the original shape has been recovered, these materials lose their shape reversibility. On the other hand, TWSMEs are able to switch between original and temporary shape several times depending on the trigger with which they are stimulated. Multiple SMEs show two or more than two temporary shapes in addition to the original shape and their transition is driven by one or more than one triggers.

As cited above, SME of SMPs can be stimulated by different types of activating agents, which can be distinguished in chemical and physical triggers [3]. Among physically induced materials, thermally responsive SMPs are sensitive to temperature changes due to their material intrinsic glass transition temperature (Tg°) and those who show Tg° near the physiologic body temperature found the greatest interest in the biomedical field. Thermally responsive SMPs, at temperature values above Tg°, become more moldable (rubbery state) and can be deformed to a desired secondary temporary shape by application of external mechanical stress. When the temperature is then lowered below Tg° (glassy state), SMPs fixed the imposed new shape. Recovery of the original shape is possible only when material is brought back to temperature values closer to its Tg° [4].

Finally, another SMP classification is performed by form/architecture; depending on fabrication process, SMPs exhibit different types of structures that can be classified as shape memory blocks, shape memory foams, shape memory fibers (micro-/nanofibers), and shape memory films [5]. Among all, fibrous structures, due to their high surface area per volume unit, high porosity, small diameter, low density, desirable fiber orientation, and nano-architecture able to mimic native extracellular matrix (ECM), are widely studied for pharmaceutical and biomedical applications such as tissue engineering, drug delivery, and regenerative medicine [6, 7]. In literature, it is reported that SMP nanofibers (SMPNs) showed enhanced shape memory effect and faster shape recovery rate compared to SMP films; this behavior is probably due to the quicker fiber heating/cooling rate due to their large surface area compared to films obtained by solvent casting. Moreover, many other fiber parameters such as fiber diameter, porosity, orientation, and morphology can influence their SME, and for this reason, they must be optimized [2, 8–10].

Considering the final biomedical applications of SMPNs, many aspects must be taken into account such as material biocompatibility, degradation rate, mechanical properties, and sterility remembering that SME must not be altered.

Sterility is a mandatory requirement for each pharmaceutical product or medical device to be implanted in the human body. Sterilization process must guarantee scaffold structural and biochemical properties maintaining their intended purposes also post-sterilization [11]. Gamma irradiation is one of the most widely used techniques for the sterilization of biodegradable polymeric materials that are sensitive to other officially approved sterilization methods such as heat sterilizations. The technique involves the use of gamma rays from a source of radioisotopes, such as Cobalt-60 that interacts with matter through the formation of ion pairs, with the expulsion of an electron, resulting in the formation of ROS (reactive oxygen species). The formation of ROS causes several effects at biological level, such as DNA strand rupture, and cellular damages that allow the inactivation of bacteria (Gram + and Gram −), molds, yeasts, most viruses, and some bacterial spores [11].

The radiation dose recommended by the European Pharmacopoeia is 25 kGy; other doses are allowed, provided the sterilization process is validated.

A sterilization process should guarantee 10^-6 sterility assurance level (SAL) is reached [12].

Gamma irradiation is advantageous for a lot of polymeric materials because it operates at low temperatures and in short times, and no quarantine of the sterilized material is required. However, polymers subjected to gamma irradiation can undergo chemical, mechanical, and morphological changes, such as polymer degradation by cross-linking, chain scission, or both. Thus, the process should be investigated before its application to a new polymeric material. For example, irradiation causes a reduction in Tg° values in those polymers for which irradiation leads to molecular weight reduction, because cleavage at the chain level results in increased molecular mobility [13, 14].

In this work, a statistical approach through design of experiment (DOE) was used to define the correlation among those process parameters affecting copolymer poly-l-lactide-co-poly-ε-caprolactone (PLA:PCL 70:30) SMPN ability to maintain rolled fixed shape (Rf%) and recover original shape (Rr%) upon temperature cycle treatment. PLA:PCL copolymer was selected due to its good properties of biocompatibility, biodegradability, and Tg near the body temperature (Tg = 32–42 °C). This last property allows to optimize a temperature-induced shape memory treatment and is able to exploit its action at physiologic temperature and it is required when performed on cell-seeded scaffolds. Moreover, PLA:PCL is a medical grade copolymer and is FDA-approved for surgical implants and drug delivery devices for tissue engineering and regenerative medicine applications [15].

Suitable electrospun scaffold was selected from DOE design and its Rf% and Rr% were theoretically evaluated using a mathematical approach. SMPN sterilization using gamma irradiation was performed and its effect on polymer shape memory effect was evaluated. Subsequent morphological (SEM), chemical-physical (GPC and DSC), mechanical (uniaxial tensile tests), and biological (cell viability and adhesion) characterizations were performed.

Thanks to the ability to fix a temporary shape, PLA:PCL SMPNs could be useful in the biomedical field to carry out a minimally invasive surgery implantation which can improve outcome of some invasive surgery and reduce surgical complications, meanwhile supporting cell adhesion and proliferation for new tissue ingrowth [2, 16].

## Materials

Copolymer poly-l-lactide-poly-ε-caprolactone (PLA-PCL) 70:30 M ratio (Resomer LC 703 S – Mw 160.000 kDa) was obtained from Evonik Industries (Evonik Nutrition & Care GmbH, 64,275, Darmstadt). Methylene chloride (MC), *N*,*N*-dimethylformamide (DMF), and tetrahydrofuran (THF) analytical grade solvents were supplied by Sigma Aldrich (Merck KGaA, Darmstadt, Germany) and used without further purification.

For cell assay, Normal Human Dermal Fibroblast (NHDF; adult skin) (34,766) cell line was used. Dulbecco Modified Eagle’s Medium (DMEM), supplemented with 4.5 g/L glucose and l-glutamide, and minimum essential medium (αMEM) from Gibco (Milano, Italy) were used for in vitro cell expansion. 3-(4,5-Dymethiltiazol-2-y)-2,5 diphenyltetrazolium bromide (MTT), 4′,6-diamidino-2-phenylindole dihydrochloride (DAPI), and LIVE/DEAD cell double staining kit purchased from Sigma Aldrich (Merck KGaA, Darmstadt, Germany) were reagents for biological characterization.

Glutaraldehyde (GA), paraformaldehyde (PFA), sodium cacodylate buffer (SCB), and dimethyl sulfoxide (DMSO) for cell assays were purchased from Sigma Aldrich (Merck KGaA, Darmstadt, Germany). Absolute ethanol (EtOH) 100% and hexadimethyldisilazane HDMS pure grade analytical grade were used.

## Methods

### DOE approach

As explained in the “Introduction” section, shape memory effect of SMPNs is influenced by nanofiber properties that are correlated to manufacturing process. In case of electrospinning, the main parameters that influence nanofiber outcome are (i) polymer concentration (wt/v%), (ii) needle size (Gauge), and (iii) spinning time (min).

In this work, a DOE approach was used to well define process parameters affecting SME of polymeric electrospun nanofibrous scaffolds. Three input factors (*x*_1_, *x*_2_, and *x*_3_) and two output levels (*y*_1_ and *y*_2_) were selected to plan a Central Composite Design (CCD 2^3^ + 3).

**Fig. 1:**
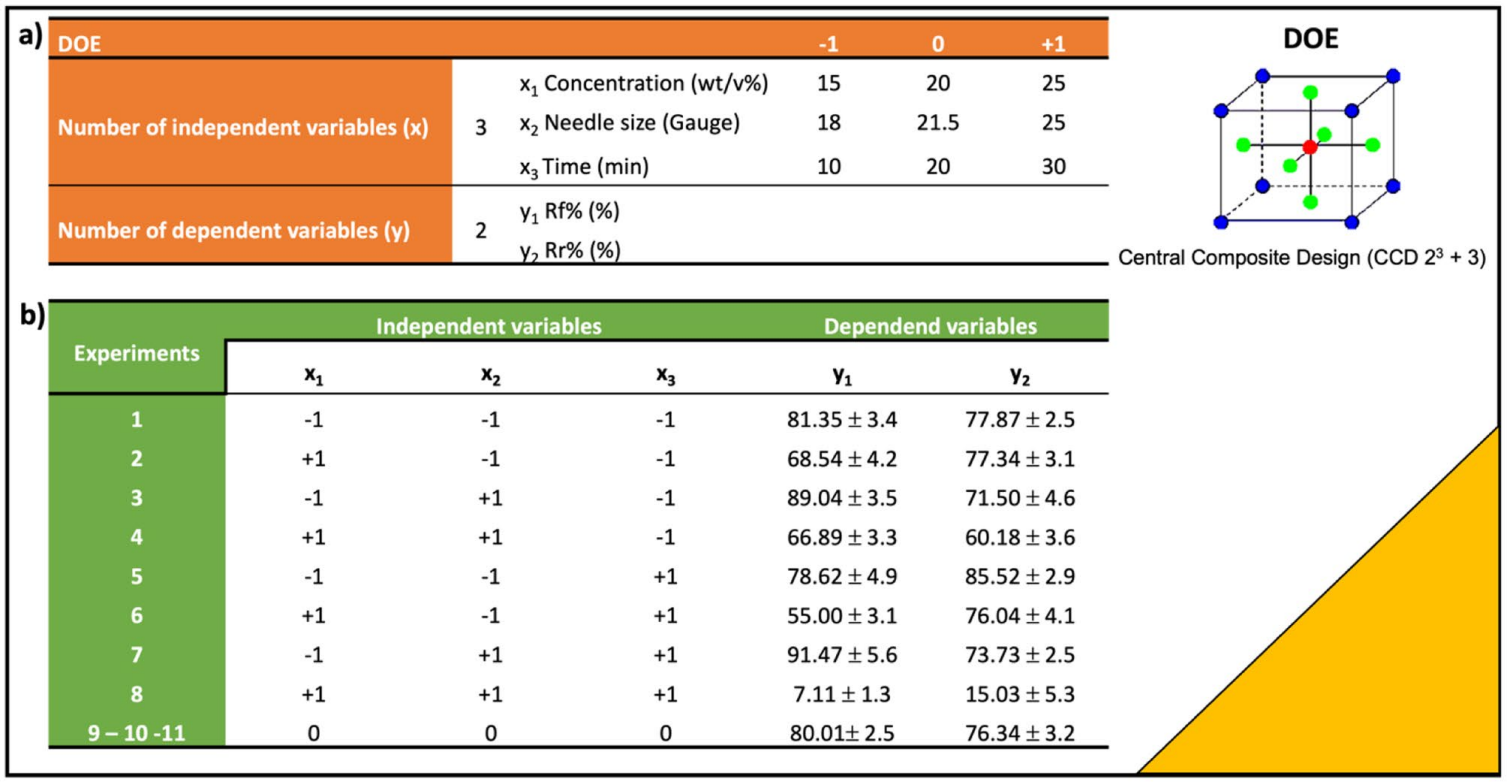
a DOE (CCD 2^3^ + 3) set up and minimum (− 1), maximum (+ 1) and intermediate (0) values of independent variables (*x*) concentration (*x*_1_), needle size (*x*_2_), and spinning time (*x*_3_). **b** Experimental design and results obtained for dependent variables *y*_1_ (Rf%) and *y*_2_ (Rr%)

The number of independent variables *x* was defined by the number of variables that can influence the final results *y*. The selected independent variables (*x*) were polymeric solution concentration (w/v %), needle size (Gauge) used in electrospinning process, and spinning time (min). Value ranges (from minimum to maximum) for each variable are reported in Fig. 1a (“Results” section). Minimum and maximum variable values were defined on the basis of previous experimental works of the same authors [17]. A total of 11 experiments were planned by DOE, matching minimum, maximum, and intermediate independent variable values. In this DOE (CCD), the experiment at the central point of experimental of range was replicated three times.

Dependent variables (*y*_1_ and *y*_2_) are the responses that define the quantifiable result of the experiments. For the setup, DOE Rf% (*y*_1_) and Rr% (*y*_2_) were selected as response variables; Rf% indicates the scaffold ability to fix new temperature-induced shape, while Rr% refers to scaffold ability to recover primary shape.

A statistical significance level of 5% was considered. Coefficient of determination (*R*^2^) was evaluated for both dependent variables. *R*^2^ is a percentage value between 0 and 100% that describes how well the model fits to the experimental data; values closer to 100% mean that the model has a perfect fit.

ANOVA analysis was performed to statistically validate results obtained from the DOE method.

Each analysis was performed in triplicate and the average data were used for DOE processing. Aexd.net software was used to create DOE (Aexd.net is a product of Alleviating Science BV, Innervate Services BV, and 043WEB Webdesign Maastricht).

### Electrospun nanofiber preparation

Poly-l-lactide-co-poly-ε-caprolactone 70:30 was used due to its biocompatibility, biodegradability, and approval by regulatory agencies (namely EMA and FDA) for use in the human body. Polymeric solutions at different concentrations (15% w/v, 20% w/v, and 25% w/v) were prepared using a solvent blend of MC and DMF 70:30 ratio. Solvent ratio had been optimized in a previous work considering solvent dielectric constant and boiling points [17].

Electrospinning setup NANON-01A equipped with dehumidifier (MEEC instrument, MP, Pioltello, Italy) was used to obtain electrospun fibers. Polymeric solutions were loaded in 5-mL syringes (Luer Lock syringe, DB) connected to a Teflon tube that is connected to a metallic spinneret. Electrospinning process parameters were set up in a previous work and used as follows: voltage (30 kV), flow rate (0.5 mL/h), needle collector distance (15 cm) [18]. A metallic plate collector was used to collect dry electrospun fibers. Polymeric solutions were electrospun maintaining constant values of temperature (25 ± 3 °C) and relative humidity (30 ± 4%).

The polymeric scaffolds obtained underwent shape memory treatment and Rf% and Rr% (*y*_1_ and *y*_2_ dependent variables) data were further processed by DOE.

### Shape memory treatment

Electrospun nanofibrous scaffolds were cut in a rectangular shape (2.5 × 1.5 cm) and treated by a temperature circuit above and below Tg° to obtain shape memory polymers nanofibers (SMPNs). The two most important parameters influencing shape memory property are temperatures and treatment time. These two parameters were optimized in a previous work in which the best combination between heating/cooling temperatures and soaking time in PBS buffer (pH 7.4) was identified [19]. The first heating step is the most important because the polymer chains must be brought from glassy to rubbery state in order to be easily moldable in a temporary rolled-up configuration C2 (see Fig. 4, “Results” section). However, since this procedure should be carried out on engineered scaffolds, cell sensitivity to temperatures above 37 °C and incubation outside their own culture medium were taken into account when fixing the shape memory–inducing protocol.

To fix a scaffold in the temporary induced shape, temperature should be set below the polymer Tg° (T < Tg°) in order to bring back the polymer chains in a glassy state. For this step, time was fixed at 10 min and temperature was 5 °C. Furthermore, it is known that temperatures around 5 °C do not damage cells; in fact, this temperature is used to store and transport organs [20]. Finally, to recover the original shape, the temperature for the third heating step was set at 37 °C because it mimics the physiological temperature of the human body where ideally the scaffold will be used, and it is slightly above polymer Tg°.

Briefly, electrospun scaffolds (in the original flat conformation C1) were immersed in a first heated bath filled with PBS (phosphate buffer saline, pH 7.4) at 40 °C for 10 min. Subsequently the scaffolds were retrieved and rolled around a 2-mm steel bar. The rolled-up scaffolds were cooled down by soaking them in a cold PBS bath at 5 °C for 10 min.

The scaffold ability to maintain temperature-induced rolled-up configuration (C2), when brought back at room temperature (Rf%), was calculated by the following equation (Eq. (1)):

1 $$Rf (\%) = 1 - [(\varepsilon f - \varepsilon i) / \varepsilon f] \times 100$$

where *ε*_*f*_ is the scaffold rolled shape registered after cold treatment (C2), and *ε*_*i*_ is the standard scaffold rolled shape that corresponds to diameter of steel bar = 2 mm.

The return to the original flat configuration (C1r) was performed dipping the scaffold in a PBS bath at 37 °C (corresponding to body physiologic temperature). When temperature was brought back to values close to copolymer Tg°, the scaffold spontaneously returned, in a certain percentage, to its original shape C1(*ε*_0_).

The scaffold ability to recover primary shape is defined by Rr% (Eq. (2))

2 $$Rr (\%) = (\varepsilon p / \varepsilon 0) \times 100$$

where *ε*_*p*_ represents the recovered conformation to *ε*_0_ primary shape obtained after 37 °C heat-induced recovery (C1r).

The two equations described above were used to determine Rf% and Rr% for each scaffold tested. Each analysis was performed in triplicate using a digital millesimal caliper and ImageJ software for measurements, and data are reported as values of Rf (%) and Rr (%) with standard deviation (± SD).

Rf% and Rr% values were inserted in the DOE model and the results obtained allowed to predict and select the shape memory scaffold that showed the highest values of Rf% and Rr% after SMT. The SMPNs selected were further characterized and their cellularization was investigated in order to achieve engineered shape memory polymer nanofibers (E-SMPNs).

### Sterilization by gamma irradiation

Electrospun samples were cut in a rectangular shape (2.5 × 1.5 cm) and placed in sterile falcon tubes inert to gamma radiation (Corning Incorporated, Life Sciences, 836 North St. Tewksbury, MA 01,876).

Gamma irradiation treatment was carried out at applied nuclear energy laboratory (LENA), University of Pavia, using a Cobalt-60 energy source for 7 days to achieve a 25 kGy radiation dose (3.5 kGy per day dose rate). Sterilization was performed at controlled room temperature (25 ± 3 °C). The 25 kGy applied sterilization dose is the one suggested by the EMA guideline on sterilization of medicinal products, active substances, excipients, and primary containers. The sterilization process was validated in a previous work [21, 22].

After gamma irradiation, polymeric electrospun scaffolds underwent shape memory treatment and their shape memory properties were verified and compared to not gamma-irradiated electrospun scaffolds.

Rf% and Rr% values were evaluated using Eqs. (1) and (2). Analyses were performed in triplicate and data reported as average values ± SD.

### Morphological characterization

Morphological characterization of selected SMPN samples before and after gamma irradiation was performed by scanning electron microscopy (SEM Zeiss EVO MA10 Apparatus, Carl Zeiss, Oberkochen, Germany) on gold-sputtered samples.

Images were obtained at 3.0 kx magnification and then processed by ImageJ software, a digital image processing computer program supported by standard image processing functions [23].

### Physical–chemical characterization:

#### Molecular weight analysis by gel permeation chromatography (GPC)

Gel permeation chromatography (GPC) was performed to evaluated molecular weight (Mw) and molecular number (Mn) of PLA:PCL SMPNs after gamma irradiation.

Analyses were performed using Agilent Technologies 1260 Infinity GPC apparatus, a chromatographic system composed by a precolumn (Agilent GPC/SEC Guard Columns) and three Ultrastyragel columns connected in series (7.7 × 250 mm each with different pore diameters of 104 Å, 103 Å, and 500 Å), a pump (Agilent Technologies 1260 Infinity), an IR detector (Agilent Technologies 1260 Infinity), and a data handling software (OpenLab and Cirrus).

PLA:PCL pristine powder and non-irradiated and gamma-irradiated PLA:PCL electrospun nanofibrous scaffolds were solubilized in THF and the solutions obtained (1 mg/mL) were filtered with a Fluoropore 0.45-μm (Millipore) filter before being injected in GPC; THF was the mobile phase and constant flow rate was set at 1 mL/min.

#### Differential scanning calorimetry

Differential scanning calorimetry (DSC) analysis was performed on powder raw materials (PLA:PCL 70:30) and on electrospun scaffolds before and after gamma irradiation. Calorimetry is commonly used to quantify amorphous or crystalline domains of semi-crystalline polymers and to monitor phase transitions and phase transition temperature, i.e., Tg° [24]. DSC Q2000 apparatus interfaced with a TA 5000 data station (TA Instruments, New Castle, DE, USA) was used. The instrument was calibrated using ultrapure indium (99.999%; melting point = 156.6 °C; melting enthalpy = 28.54 J·g^−1^) as standard. The samples (about 10–12 mg) were scanned at heating rates of 5 K·min^−1^ under nitrogen flow (45 mL·min^−1^) in open standard aluminum pans.

### Mechanical characterization

Mechanical properties of electrospun nanofibrous scaffolds before and after gamma irradiation were analyzed using axial tensiometer MARK-10. Electrospun scaffolds were cut in dog bone shape (0.4 cm × 2.5 cm) using a die-cutting machine, according to ISO standard 17,025:2017. This shape assures analysis reproducibility allowing the force to be applied always in the same sample point.

Dog bone–shaped scaffolds were subjected to tensile test at room temperature (25 ± 3 °C). A tensile deformation (stress–strain analysis) was performed. Uniaxial stress was applied to stretch the material and a relationship established with the resulting strain. Loading velocity was 5 mm/min because, according to the literature, this speed is used for soft tissue material characterization [25, 26].

Mechanical tensile test gives values of Young’s modulus (measure of intrinsic material stiffness) that is a measure of the ability of a material to withstand changes in length when undergoing lengthwise tension or compression [27]. Values of Young’s modulus were obtained as slope of the linear region (elastic region) in the stress (*σ*)/strain (*ε*) graph. Young’s modulus is expressed also by Eq. (3).

3 $${{Young's}}\;{Modulus}= \frac{\sigma }{\varepsilon }$$

Young’s modulus is inversely proportional to elongation (*ε*). Elongation % was obtained by Eq. (4) and represents elongation achieved by the matrix before breaking.

4 $${Elongation }\;(\varepsilon )\; \%=\frac{\Delta L}{L} \times 100$$

where Δ*L* is the change in length while *L* is the initial length [28].

Elongation is visible in the plastic region of stress (*σ*)/strain (*ε*) graph. Some factors impact elongation such as fiber orientation; if fibers are less oriented, sample tend to exhibit greater degree of elongation [29].

### Shape memory scaffold cellularization

Electrospun scaffolds and gamma-irradiated electrospun scaffolds were incubated with NHDF and biologically characterized. The gamma-irradiated electrospun scaffolds did not undergo any further treatment before being cell seeded, whereas the non-irradiated electrospun scaffolds were sanitized by treatment with EtOH solutions under laminar hood. Scaffold sanitization was performed as follows: the scaffold samples fixed in CellCrown systems were immersed in 85% v/v EtOH solution for 20 min and then for another 15 min in a 70% v/v EtOH solution. EtOH solution was removed and the scaffolds were rinsed with sterile PBS added with 2% penicillin/streptomycin (P/S) and left under UV light irradiation overnight.

A total of 100.000 NHDF/100 μL were seeded on each scaffold and directly into the wells (control) in complete DMEM medium and incubated at 37 °C, 5%CO_2_ for 7 days, replacing it twice a week.

After 7 days of incubation, all cellularized scaffolds underwent SMT protocol explained here above (see the “Shape memory treatment” section) and then soaked in DMEM cell medium at 37 °C where they recovered their primary shape, and incubated at 37 °C, 5% CO_2_ for the following 24 h (8 days of total incubation time).

Engineered SMPNs and NHDF cell controls underwent cell viability tests (colorimetric MTT test and LIVE/DEAD Assay) and imaging characterization (colorimetric DAPI assay and SEM).

### Biological characterization

#### MTT

MTT (3-[4,5-dimethylthiazol-2-yl]-2,5-diphenyltertrazolium bromide) is a colorimetric assay used to determine cell viability. Cell culture medium was removed from cellularized scaffolds and NHDF cells used as control. Afterwards, the samples were washed twice with PBS sterile solution. PBS was removed and 100 µL of MTT solution (5 mg/mL) in PBS was added to each sample; furthermore, 900 µL of PBS was added to cover all samples. Samples treated with MTT solution were incubated at 37 °C, 5% CO_2_ for 2.5 h, and then recovered for following analysis.

SMESs were removed from the wells and placed in glass beakers, where they were treated with 1 mL of THF in order to completely dissolve the polymer matrix and lyse the membranes of cells trapped in the scaffolds. Cell membranes lysis causes the release of formazan crystals deposited inside the cells and their subsequent dissolution results in purple coloring able to be quantified by spectrophotometric analysis. The samples were subjected to magnetic stirring for 60 min at 300 rpm to guarantee sample homogeneity. Spectrophotometric analysis of scaffold solution was carried out at 570 nm using quartz cuvettes (6705 UV/Vis Spectrophotometer, Single Mobile Holder, Jenway) while cell samples were transferred in a 96-multiwell plate (100 µL each well) and analyzed using a microplate reader at 570–690 nm (Microplate Photometer MPP-96, HiPo Biosan, Nebikon, Switzerland).

The test was performed twice in triplicate and data are reported as cell viability % with SD values.

#### LIVE and DEAD assay

LIVE/Dead assay is used to specifically distinguish live and dead cells. The technique is based on two fluorescent probes: calcein-AM and propidium iodide.

LIVE/DEAD kit was used to analyze by imaging either live or dead cells of cell control (Crt +) and SMESc after 8 and 10 days of incubation.

The two fluorescent reagents permit to discriminate population of live cells (green) from the dead cell population (red). Green-fluorescent calcein-AM is able to pass through membrane of live cells and interact with intracellular esterase while red-fluorescent ethidium homodimer-1 binds dead cells that lost plasma membrane integrity.

Fluorescent microscope Leica DM IL LED-FLUO (Leica Microsystems Srl, Buccinasco, MI, I-20090, Italy) equipped with lens 10 × , 20 × , and 40 × was used.

The samples were washed from DMEM cell medium using PBS sterile solution. Previously, solutions contained in LIVE/Dead Cell Imaging Kit, Live Green (comp. A) and Dead Red (Comp. B), were mixed to create 2 × working solution. An equal volume (200 μL) of 2 × working solution was dropped into samples and left for 15 min at 25 °C protected by light. Samples were then washed with PBS and mounted on microscope glass slides covered by a coverslip.

A fluorescent microscope was equipped with lens and set at excitation (494 nm) and emission (517 nm) wavelength for calcein-AM and excitation (528 nm) and emission (617 nm) wavelength for ethidium homodimer-1 to achieve images at 20 × magnification.

#### DAPI staining assay

DAPI (4,6-diamidino-2-phenylindole) is a dye that is used as nuclear staining technique because it can bind specifically to DNA, preferentially at level of A-T-rich regions.

DAPI staining was used to detect cells binding their nucleus. Control cells (Ctrl +) and SMESs after 8 days (meaning 24 h after SMT for engineered scaffolds) were fixed with DAPI solution for imaging characterization using fluorescent microscopy.

Cell medium was removed from NHDF control and E-SMPN samples, and washed with PBS. A 0.4% v/v glutaraldehyde (GA) solution (1 mL) was dropped on each sample for 15 min in order to fix cells. GA solution was removed and samples washed twice with PBS in order to remove all traces of GA. Cell membrane permeabilization was obtained through addition of a solution composed by 0.1% Triton-X in PBS that promoted DAPI entrance into the cell nucleus. After 10 min of incubation, the samples were washed again with PBS to remove excess of Triton-X, and then treated with 200 μL of PBS solution with DAPI (300 μg/mL) for 30 min protected by light. After staining, samples were further washed with PBS to remove DAPI surplus and mounted on microscope glass slides covered by a coverslip (22 × 22 mm, 0.17-mm thickness).

The fluorescent microscope was equipped with lens and set at excitation (358 nm) and emission (461 nm) wavelength to achieve images at 20 × magnification.

#### Scanning electron microscope analysis

A scanning electron microscope (SEM) Zeiss EVO MA10 apparatus (Carl Zeiss, Oberkochen, Germany) was used for morphological characterization. Images were obtained at 3.0 kX magnification and then processed by ImageJ software.

The samples for SEM analysis were prepared as follows: NHDF control cells and E-SMPNs at 8 days of incubation, after cell medium removal and sodium cacodylate buffer (SCB) (0.1 M, pH = 7.4) washing, were fixed with 2% w/v GA–2% w/v PFA solution in SCB (pH = 7.4) for 30 min and incubated at 37° C. GA was removed with two more washing steps using SCB solution. Following dehydration was carried out with aqueous ethanol solutions at increasing concentrations: 30, 50, 70, and 90% up to anhydrous ethanol (100%); each passage lasted 7 min and the samples were kept in a refrigerator between each passage. Then, the samples were washed twice with a 50:50 mixture of anhydrous EtOH 100% and hexadimethyldisilazane (HDMS) (first time for 30 min and second time for 20 min). For the last step of dehydration protocol, pure HDMS solvent was used for 30 min.

Samples were left under laminar flow hood overnight to completely dry and remove residual solvents.

Dry samples (cell control and SMESs) were gold-sputtered before SEM analysis.

## Results

### DOE approach

DOE was completed putting experimental results (Rf% and Rr%) obtained from treated SMPNs in the design table; every row in the table represents one treatment in the experimental design. The results of Rf% and Rr% values were evaluated for each scaffold that underwent SMT protocol and reported in Fig. 1b.

The table shows that the 15% w/v and 20% w/v solutions result in the highest Rf% values, whereas the solutions obtained using 18G needle seem to have better Rr% values.

SMPNs obtained with the highest polymer concentration solutions (25% w/v) did not guarantee a suitable shape memory effect.

**Fig. 2:**
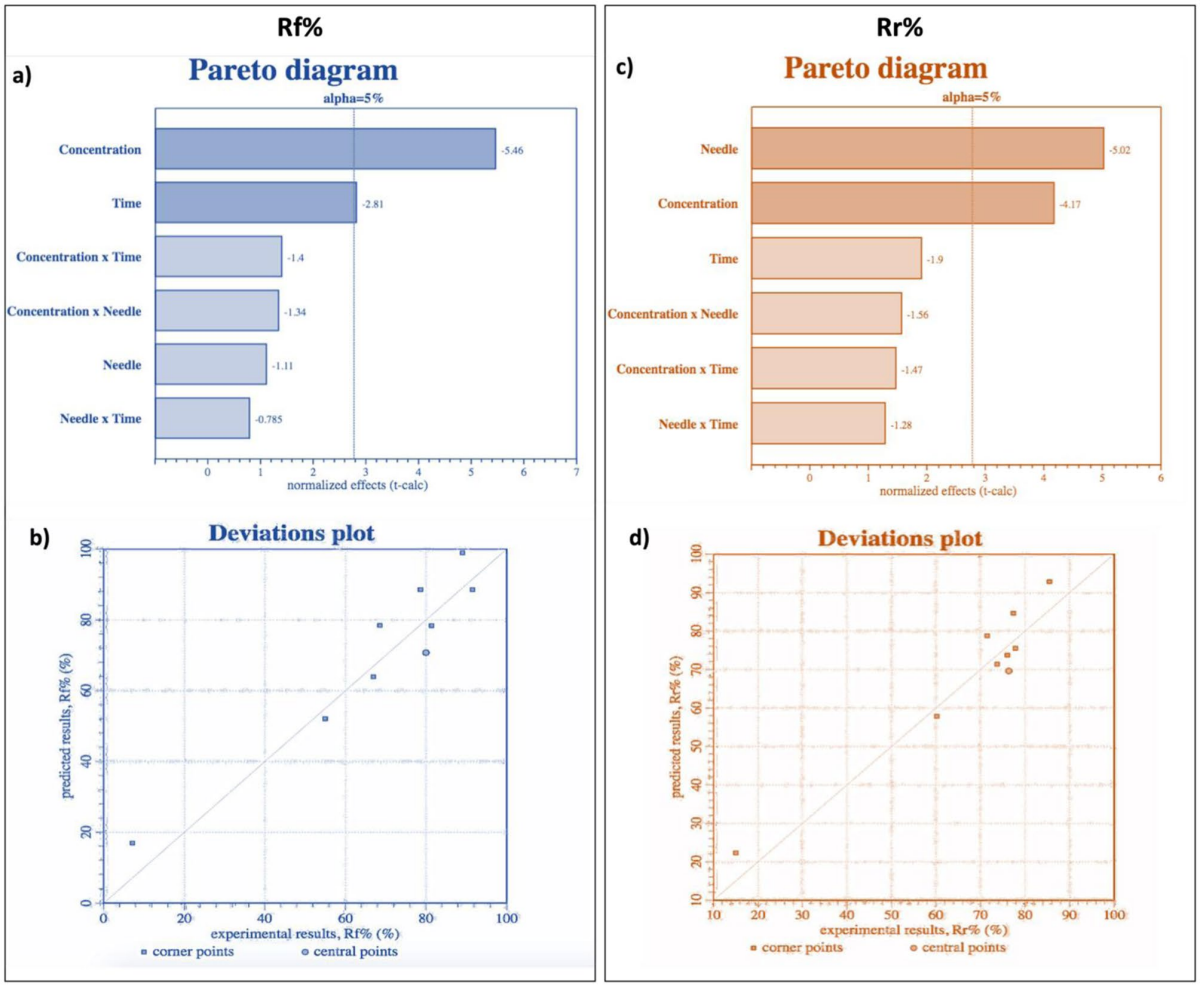
**a** Pareto diagram for Rf% (*y*_1_) dependent variable (*p* value < 0.0001). **b** Deviation plot for Rf% (*y*_1_) dependent variable (*R*^2^: 87.58%). **c** Pareto diagram for Rr% (*y*_2_) dependent variable (*p* value < 0.0001). **d** Deviation plot for Rr% (*y*_2_) dependent variable (*R*^2^: 89.89%)

The results obtained allowed to identify which independent variable (*x*) correlation most influences the desired outputs for Rf%. Pareto diagrams reported in Fig. 2a show the absolute normalized effects (*t*-values) ordered by magnitude. Factors with an absolute *t*-value higher than a tabulated *t*-value for given a significance level (5%) and degree of freedom (shown as a vertical line) resulted statistically significant. In this case, concentration of polymeric solution (w/v%) and spinning time (min) are the parameters that mainly influence Rf%.

Deviation plot reported in Fig. 2b visually compares the experimental Rf% results (horizontal axis) and the results predicted by the DOE model (vertical axis), for each sample, showing which result the model predicts (vertical axis) for each experimental result (horizontal axis). Points closer to the plot diagonal line are the more reliable in the model, at least within the range studied values. A Rf% coefficient of determination *R*^2^ of 87.58% was obtained.

The data obtained allowed to identify independent variable (*x*) correlation that most influenced the desired outputs for Rr%. Pareto diagrams reported in Fig. 2c show the absolute normalized effects (*t*-values) ordered by magnitude. Needle size (*G*) and concentration of polymeric solution (w/v%) are the two parameters that most influence Rr%. Deviation plot reported in Fig. 2d visually compares the experimental Rr% results obtained, and the results predicted by the DOE model, for each sample analyzed. A Rr% coefficient of determination *R*^2^ of 89.89% was obtained.

**Fig. 3:**
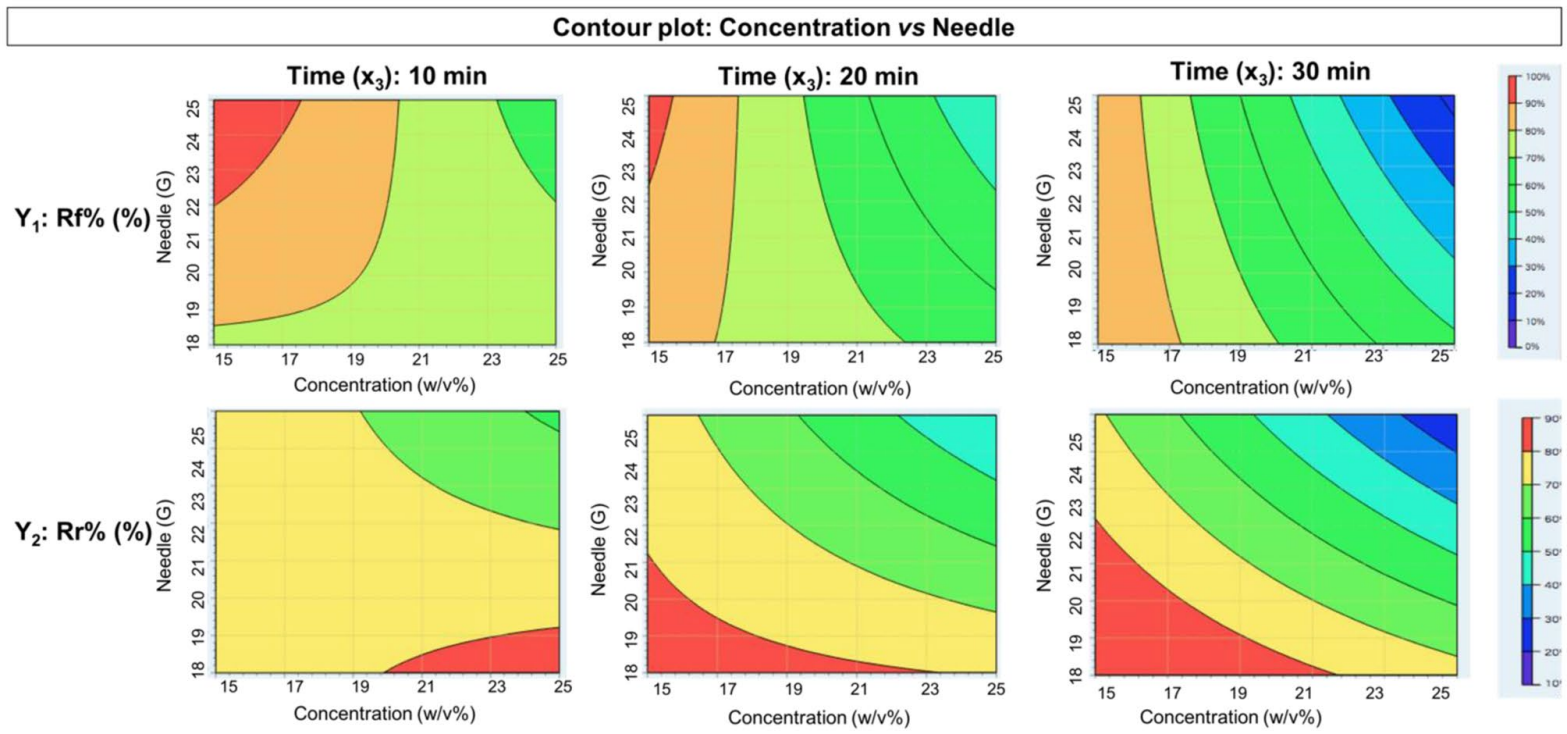
Contour plot concentration vs needle at three different times for dependent variable *y*_1_ Rf% and *y*_2_ Rr%

The data were also presented using contour plot, a two-dimensional display of the dependence of the response variables Rf% and Rr% (Fig. 3) versus the two independent variables. The highest values of response variables correspond to red color (90–100%). The value ranges for each color are shown in the color bar in Fig. 3.

Using contour plots, it is possible to predict scaffold Rf% and Rr% of PLA:PCL solutions in different ranges of concentrations between 15 to 25% w/v, needle between 18 and 25 G, and at different spinning times (10, 20, and 30 min). A mathematical model was obtained from DOE design, able to calculate theoretical Rf% and Rr% starting from specific values of PLA:PCL concentration, needle, and spinning time. Predictive equations obtained for Rf% value (Eq. (5)) and Rr% (Eq. (6)) are reported here below.

5 $$\begin{aligned}\mathrm{Rf\%}=&\; -168.5+10.839786\;\mathrm{ Concetration}+11.91\;\mathrm{ Needle}\\&+5.8851071\;\mathrm{ Time}-0.50057143\;\mathrm{ Concentration }\\&\times \mathrm{ Neelde}-0.18255\;\mathrm{ Concentration }\times \;\mathrm{ Time}\\&-0.14671429\;\mathrm{ Needle }\times \mathrm{ Time}\end{aligned}$$

6 $$\begin{aligned}\mathrm{Rr\%}=&\;123.5+10.031571\;\mathrm{ Concentration}\\&+8.6517857\;\mathrm{ Needle}+6.1426071\;\mathrm{ Time}\\&-0.42864286\;\mathrm{ Concentration }\times \mathrm{ Needle}\\&-0.14082500\;\mathrm{ Concentration }\times \mathrm{ Time}\\&-0.17596429\;\mathrm{ Needle }\times \mathrm{ Time}\end{aligned}$$

The parameter combination PLA:PCL solution 15% w/v, 22G needle, and 20 min spinning time was selected as suitable and the values of Rf% = 89.31% and Rr% = 79.04% were obtained applying Eqs. (5) and (6). Following characterization was performed on the PLA:PCL selected scaffold, prepared using the electrospinning process parameters set above, i.e., voltage (30 kV), flow rate (0.5 mL/h), needle collector distance (15 cm) (see the “Electrospun nanofibers preparation” section).

### Shape memory treatment

**Fig. 4:**
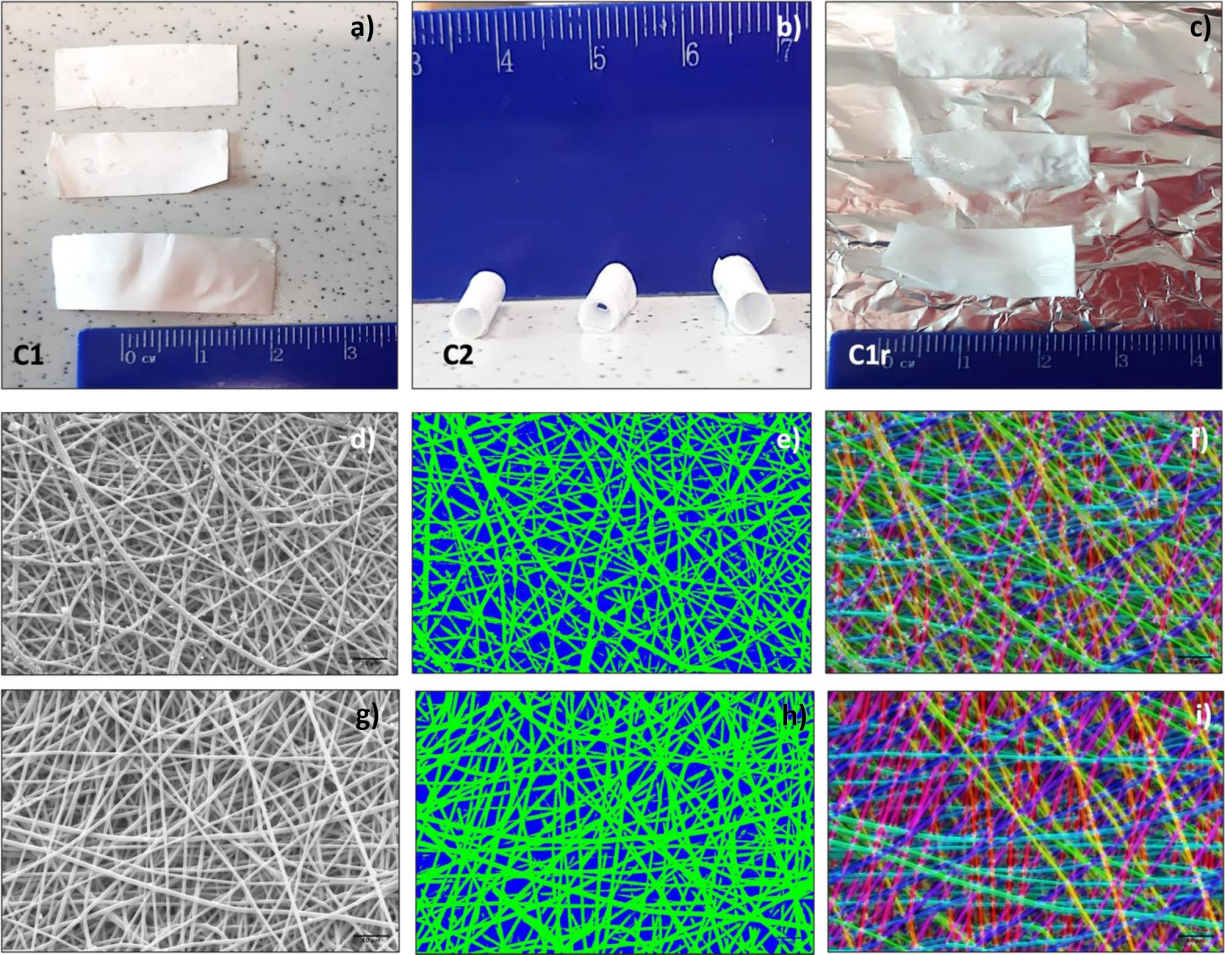
Shape memory treatment (SMT). **a** Scaffold configuration before treatment, C1. **b** After first heating treatment (T_1_ > Tg°) and cooling bath (T_2_ < Tg°), scaffolds maintained rolled shape (configuration C2) and **c** after temperature treatment close to Tg° (37° C), scaffolds recovered original shape (configuration C1r). Morphological characterization of 15% PLA-PCL electrospun nanofibers obtained using 22 G needle for 20 min spinning time before SMT. **d** SEM image. **e** Fiber porosity analysis. **f** Fiber orientation analysis. Morphological characterization after SMT. **g** SEM image. **h** Fiber porosity analysis. **i** Fiber orientation analysis

SMT protocol was optimized in a previous work and used to obtain Rf% and Rr% that were further processed with DOE design table (data reported in Fig. 1). SMT was performed on the selected PLA:PCL electrospun scaffolds (15% w/v, 22G needle, and 20 min spinning time) and SMT steps are schematized in Fig. 4, showing how the flat shape of scaffold (C1) kept its conformation after rolling up and temperature treatment below polymer Tg° (C2), and eventually recovered its original shape after treatment at 37 °C (T3 above polymer Tg°). Scaffold original shape recovery (C3) is induced by heating treatment without applying any mechanical force. The experimental values of Rf% and Rr% obtained, i.e., Rf% = 88.21 ± 3.4% and Rr% = 81.12 ± 5.3%, were in line with those obtained from theoretical approach, and confirmed the validity of mathematical model.

### Morphological characterization

Morphological characterization of the selected PLA:PCL electrospun scaffolds (15% w/v, 22G needle, and 20 min spinning time) before and after SMT was performed by SEM. Images obtained were processed using ImageJ software to evaluate fiber dimension and porosity (Fig. 4).

The nanofibers before SMT showed diameter in a nanometer size range (770 ± 140 nm).

Porosity % obtained through ImageJ processing was 1.23 ± 0.9% and pores area was 3.47 ± 2.97 μm^2^. After SMT, fibers did not show significant variation keeping a fiber diameter size range of 760 ± 96 nm, porosity 2.76%, and pores area of 3.36 ± 3.28 μm^2^. Moreover, no change in fiber network structure was detected after SMT treatment (see Fig. 4d–i).

Gamma-irradiated fibers were also characterized for their morphology by SEM analysis and no significant modification in terms of fiber diameter was detected (data reported in a previous work [30]).

### Physical–chemical characterization

#### Effect of gamma irradiation on polymer shape memory property

The selected PLA-PCL electrospun scaffolds were sterilized using gamma irradiation and then characterized to evaluated if after irradiation the scaffolds were still able to guarantee suitable shape memory effect, or any change raised from the sterilization treatment. Gamma-irradiated scaffolds underwent the optimized SMT and values of Rf% and Rr% were evaluated. Irradiated SMPNs showed higher values of Rf% = 93.21 ± 2.7% and Rr% = 86.12 ± 3.3% compared to non-irradiated scaffold.

#### Molecular weight analysis by gel permeation chromatography

**Fig. 5:**
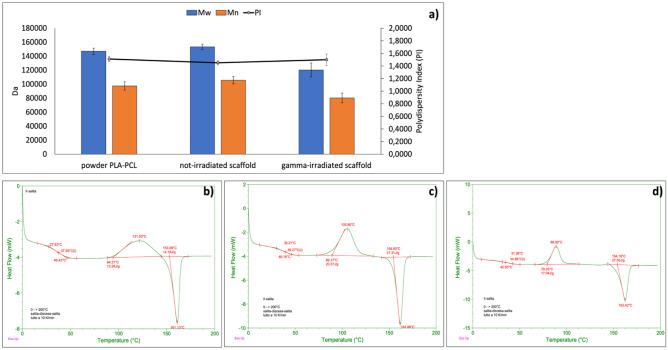
**a** Results of GPC analyses reporting Mw, Mn, and PI of PLA-PCL powder and non-irradiated and gamma-irradiated PLA-PCL electrospun scaffolds. **b**–**d** Results of DSC analysis performed on **b** PLA-PCL powder; **c** non-irradiated PLA-PCL electrospun nanofibers; and **d** gamma-irradiated PLA-PCL electrospun nanofibers

Results of GPC analyses, performed on PLA:PCL powder and non-irradiated and gamma-irradiated PLA:PCL electrospun scaffolds, are reported in Fig. 5.

The results confirmed that gamma-irradiated PLA:PCL electrospun scaffolds suffered a Mw reduction of about 21.53% and Mn reduction of about 17.55% after 25 kGy dose irradiation. Considering the sensitivity of GPC analysis is within 10% variation, the Mw and Mn reductions after irradiation are significant. Surprisingly, polydispersity index (PI) did not change significantly after irradiation (1.45 PI for non-irradiated and 1.5 PI for gamma-irradiated).

#### Differential scanning calorimetry

DSC analysis was performed to confirm Tg° values of PLA:PCL and evaluate if Tg° values modified after gamma irradiation treatment. The DSC traces of non-irradiated and gamma-irradiated PLA-PCL electrospun scaffolds are reported in Fig. 5. Copolymer PLA:PCL was purchased from Sigma Aldrich and its data sheet reported Tg° range values between 32 and 42° C. DSC analyses confirmed this Tg° value range for pristine PLA:PCL powder (Fig. 5b). Non-irradiated PLA:PCL electrospun scaffold showed a 39.27 °C Tg° (Fig. 5c), confirming that electrospinning process affects copolymer thermal behavior because it affects polymer solid state in terms of crystalline and amorphous percentages [31]. On the other hand, gamma-irradiated PLA:PCL electrospun scaffolds exhibited lower value of Tg° = 34.86 °C (Fig. 5d). This result confirms what is reported in the literature [14] and is attributed to polymer chains cleavage caused by gamma irradiation. The results of GPC analysis shown in Fig. 5a corroborated this hypothesis.

### Mechanical characterization

An axial tensile test was performed on non-irradiated and gamma-irradiated PLA:PCL electrospun scaffolds. As reported by the results of physical–chemical characterization, gamma irradiation caused a decrease of Mw, Mn, and Tg values. Consistently, the mechanical properties of scaffolds significantly changed after gamma irradiation with a significant reduction of elasticity detected in gamma-irradiated scaffolds (Table 1).

**Table 1:** Young’s modulus and elongation at break % of non-irradiated and gamma-irradiated PLA:PCL electrospun scaffolds

**Sample**	**Young’s modulus (MPa)**	**Elongation at break %**
Non-irradiated scaffolds	0.33 ± 0.03	200 ± 12.5
Gamma-irradiated scaffolds	0.71 ± 0.1	160 ± 15.6

The results reported in Table 1 show that after gamma irradiation the scaffolds become more brittle and significantly decrease their elasticity. This behavior is confirmed by an increase of Young’s modulus and a decrease of elongation at break % for gamma-irradiated electrospun scaffolds.

### Biological characterization: MTT test, LIVE and Dead assay, and DAPI

**Fig. 6:**
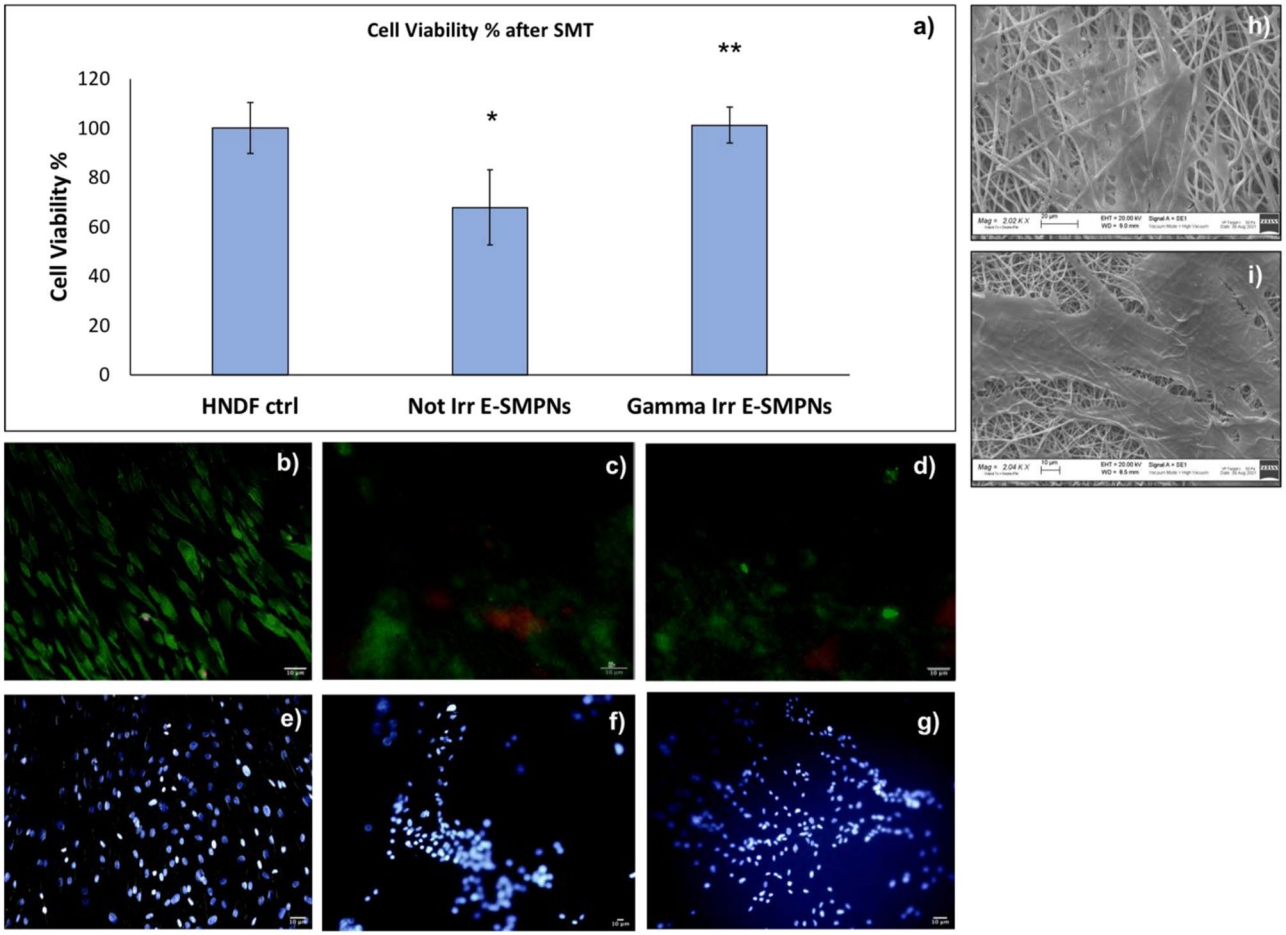
**a** Results of cell viability % determined by MTT assay on NHDF, incubated with non-irradiated and gamma-irradiated PLA-PCL electrospun scaffold after 7 days of incubation and 6 h after SMT. **b**–**g** Merge of LIVE (green)/DEAD (red) staining of Human Dermal Fibroblast after 7 days of incubation: **b** control; **c** non-gamma-irradiated shape memory electrospun scaffold; **d** gamma-irradiated shape memory electrospun scaffold. DAPI staining of NHDF after 7 days of incubation: **e** control; **f** non-gamma-irradiated shape memory electrospun scaffold; **g** gamma-irradiated shape memory electrospun scaffold. SEM images of Human Dermal Fibroblast after 7 days of incubation on **h** non-gamma-irradiated shape memory electrospun scaffold and **i** gamma-irradiated shape memory electrospun scaffold

Biological characterization was performed on non-irradiated and gamma-irradiated PLA-PCL scaffold incubated for 7 days with NHDF and then subjected to SMT. Cell viability % results are reported in Fig. 6a. PLA:PCL is a medical grade copolymer and its biocompatibility is well known. The results of MTT test showed that significantly higher cell viability was detected in gamma-irradiated engineered shape memory scaffolds (E-SMPNs; see Fig. 6a).

The result of the MTT test was confirmed by the LIVE/DEAD assay, as reported in Fig. 6b–d: live (green) cells were observed on both shape memory engineered scaffolds and higher numbers of live cells were detected on gamma-irradiated shape memory scaffold surface.

NHDF cell presence on shape memory engineered scaffold was confirmed by DAPI staining. Images are reported in Fig. 6e–g. As reported with LIVE-DEAD assay (Fig. 6b–d), DAPI staining confirmed a higher number of cells on the surface of gamma-irradiated E-SMPNs compared to non-irradiated E-SMPN scaffolds (Fig. 6g–f). Moreover, it is important to underline that after SMT, presence of NHDF cells on scaffold surface was guaranteed.

#### SEM

SEM analysis of cellularized scaffolds was performed to further evaluate the presence of NHDF cells on E-SMPNs (Fig. 6d–h).

LIVE-DEAD, DAPI, and SEM confirmed the presence of cells on the scaffolds before and after irradiation, and their vitality. Gamma-irradiated E-SMPNs (Fig. 6h, i) confirmed higher cellularization compared to non-irradiated E-SMPNs (Fig. 6h).

## Discussion and conclusion

In this work, DOE was applied as a statistical approach to define process parameters’ correlation to PLA:PCL SMPN shape memory effect. DOE is a widely used approach to optimized nano-system performance with known input variables, and it has been already applied in the field of electrospun nanofibers as reported in the literature [32, 33]. For example, Coles et al. used DOE to highlight the interaction between process parameters in manufacturing micro- and nanofibers to suit a desired application with specific material properties [34]. In our work, for the first time to the best of our knowledge, DOE was applied to optimize the parameters influencing SME of electrospun nanofibers.

Copolymer solutions were electrospun following DOE indications concerning optimization of copolymer solution concentrations (w/v%), needle size (gauge), and spinning time (min).

Nanofibrous scaffolds obtained in a flat original shape were heated up to 40 °C, which temperature is higher than copolymer Tg° as confirmed by DSC analysis. When nanofibrous scaffolds reached this temperature, they were rolled up, and then they were cooled down to a temperature of 5 °C (T° < Tg°) to fix their temporary shape. The ability to maintain fixed shape (Rf%) was evaluated for all the electrospun scaffolds prepared. Only when scaffolds were immersed in a PBS buffer at physiologic temperature (37 °C) was the original flat shape recovered at different percentage as rated by Rr% value.

DOE design highlighted which parameters most influenced Rf% and Rr% and have a significant impact on final results, showing that polymeric solutions at 15% w/v (minimum concentration values) resulted in electrospun matrices with higher Rf% and Rr% values compared to those obtained from 25% w/v solutions (maximum concentration values). This behavior might be correlated to fiber dimension which increases along with increasing polymer concentration leading to decrease the fiber surface area interacting in the heating/cooling SMT [35]. Sauter et al. demonstrated the same trend with polyetherurethane (PEU) nanofibers for which reduction of the single fiber diameter (< 100 nm) improved the shape memory performance of the scaffolds [9]. Therefore, it can be hypothesized that the shape memory property of PLA:PCL, even being an intrinsic property of stimuli responsive polymers, is also affected by the scaffold structure, i.e., fiber diameter.

A mathematical model was set up and it was demonstrated that it allowed to predict Rf% and Rr% values for PLA-PCL SMPNs. The mathematical model was applied for 15% w/v PLA-PCL electrospun scaffold obtained using 22G needle for 20 min spinning time and theoretical data (Rf% = 89.31% and Rr% = 79.04%) were confirmed by experimental results (Rf% = 88.21 ± 3.4% and Rr% = 81.12 ± 5.3%).

Since the scaffolds are intended for a biomedical application, gamma irradiation sterilization (25 kGy) was performed and SMPNs were tested to guarantee a suitable SME was kept even after gamma irradiation. The gamma-irradiated scaffolds were characterized for their physical–chemical, mechanical, and biological behavior in comparison with to non-irradiated SMPNs and resulted to keep suitable SME (Rf% = 93.21 ± 2.7% and Rr% = 86.12 ± 3.3%) with even higher values if compared to non-irradiated SMPNs. The change in SME is a consequence of a decrease in copolymer Mw (− 21.53%) and Mn (− 17.55%) that was highlighted after irradiation. Consistently, Tg° of gamma-irradiated SMPNs significantly decreased from 37.59 °C (raw material) to 34.86 °C (gamma-irradiated scaffold). Probably this slight decrease in Tg° makes the SMPNs more susceptible to heat treatment, guaranteeing a greater softening of the chains which favors both the manipulation of the transitory shape and the recovery of the original shape. Summarizing, the results are consistent and demonstrated the shape memory behaviors of the PLA:PCL scaffolds are strictly connected to physical–chemical properties of PLA:PCL and to scaffold morphology.

Gamma irradiation caused also embrittlement of the electrospun nanofibrous scaffolds; SMPNs become more brittle and lost their elasticity. This behavior is confirmed by an increasing Young modulus (0.71 ± 0.1 MPa) and a decrease of elongation at break % (160 ± 15.6%) for gamma-irradiated SMPNs. However, mechanical property values obtained after sterilization process remain within an acceptable range of values applicable to soft tissue regeneration [27].

Biological characterization performed with HNDF confirmed the biocompatibility of copolymer PLA-PCL. Cell viability % evaluated after SMT on cellularized irradiated and non-irradiated E-SMPNs showed that significant higher cell viability was highlighted in gamma-irradiated SMPNs. This result is justifiable by the fact that material stiffness influences cell proliferation. El-Mohri et al. demonstrated that bio-scaffolds with higher stiffness (23 kPa) showed better results in terms of fibroblast cell growth (%) and cell area (μm^2^) covered [36].

Moreover, other biological characterizations performed confirmed the presence of live and adherent cells on E-SMPNs.

In conclusion of this proof of concept, it is possible to affirm that DOE is a valid tool that allows the selection of the best parameters’ combination to achieve polymeric SMPNs in relation to the desired outputs. The robustness of the developed DOE approach was confirmed by the fact that theoretical Rf% and Rr% values obtained from DOE are in agreement with the obtained experimental values.

Gamma irradiation did not affect fiber morphology but influenced SMPNs Tg° value and Rf%, Rr%, and mechanical properties. Eventually, the results suggest that is important to include sterilization techniques, and the evaluation of their effect, on preliminary in vitro characterization of SMPs for biomedical applications.

## Data Availability

The datasets generated and/or analyzed during the current study are available from the corresponding author on reasonable request.

## Abbreviations

C1: Configuration 1
C2: Configuration 2
C1r: Configuration 1 recovered
CCD: Central Composite Design
DMF: *N*,*N*-Dimethylformamide
DSC: Differential scanning calorimetry
DOE: Design of experiment
ECM: Extracellular matrix
E-SMPNs: Engineered shape memory polymers nanofibers
G: Gauge
GPC: Gel permeation chromatography
MC: Methylene chloride
OWSME: One-way shape memory effect
PLA-PCL: Poly-l-lactide-poly-ε-caprolactone
Rf%: Ability to fix temporary shape
Rr%: Ability to recover original shape
ROS: Reactive oxygen species
SEM: Scanning electron microscopy
SME: Shape memory effect
SMPs: Shape memory polymers
SMPNs: Shape memory polymers nanofibers
Tg°: Glass transition temperature
THF: Tetrahydrofuran
TWSME: Two-way shape memory effect
